# Genomic and pedigree-based prediction for leaf, stem, and stripe rust resistance in wheat

**DOI:** 10.1007/s00122-017-2897-1

**Published:** 2017-04-09

**Authors:** Philomin Juliana, Ravi P. Singh, Pawan K. Singh, Jose Crossa, Julio Huerta-Espino, Caixia Lan, Sridhar Bhavani, Jessica E. Rutkoski, Jesse A. Poland, Gary C. Bergstrom, Mark E. Sorrells

**Affiliations:** 1 Plant Breeding and Genetics Section, School of Integrative Plant Science, Cornell University, Ithaca, NY 14853 USA; 2 International Maize and Wheat Improvement Center (CIMMYT), Apdo, Postal 6-641, 06600 Mexico, DF Mexico; 3 Campo Experimental Valle de México INIFAP, 56230 Chapingo, Edo, de México, Mexico; 4 CIMMYT, ICRAF House, United Nations Avenue, Gigiri, Village Market, Nairobi, 00621 Kenya; 5 Wheat Genetics Resource Center, Department of Plant Pathology and Department of Agronomy, Kansas State University, Manhattan, KS 66506 USA; 6 Plant Pathology and Plant-microbe Biology Section, School of Integrative Plant Science, Cornell University, Ithaca, NY 14853 USA

## Abstract

**Key message:**

**Genomic prediction for seedling and adult plant** **resistance to wheat rusts was compared to prediction using few markers as fixed effects in a least-squares approach and pedigree-based prediction**.

**Abstract:**

The unceasing plant-pathogen arms race and ephemeral nature of some rust resistance genes have been challenging for wheat (*Triticum aestivum* L.) breeding programs and farmers. Hence, it is important to devise strategies for effective evaluation and exploitation of quantitative rust resistance. One promising approach that could accelerate gain from selection for rust resistance is ‘genomic selection’ which utilizes dense genome-wide markers to estimate the breeding values (BVs) for quantitative traits. Our objective was to compare three genomic prediction models including genomic best linear unbiased prediction (GBLUP), GBLUP A that was GBLUP with selected loci as fixed effects and reproducing kernel Hilbert spaces-markers (RKHS-M) with least-squares (LS) approach, RKHS-pedigree (RKHS-P), and RKHS markers and pedigree (RKHS-MP) to determine the BVs for seedling and/or adult plant resistance (APR) to leaf rust (LR), stem rust (SR), and stripe rust (YR). The 333 lines in the 45th IBWSN and the 313 lines in the 46th IBWSN were genotyped using genotyping-by-sequencing and phenotyped in replicated trials. The mean prediction accuracies ranged from 0.31–0.74 for LR seedling, 0.12–0.56 for LR APR, 0.31–0.65 for SR APR, 0.70–0.78 for YR seedling, and 0.34–0.71 for YR APR. For most datasets, the RKHS-MP model gave the highest accuracies, while LS gave the lowest. GBLUP, GBLUP A, RKHS-M, and RKHS-P models gave similar accuracies. Using genome-wide marker-based models resulted in an average of 42% increase in accuracy over LS. We conclude that GS is a promising approach for improvement of quantitative rust resistance and can be implemented in the breeding pipeline.

**Electronic supplementary material:**

The online version of this article (doi:10.1007/s00122-017-2897-1) contains supplementary material, which is available to authorized users.

## Introduction

Wheat (*Triticum aestivum* L.) is one of the major food crops in the world that is constantly threatened by several biotic stresses. Among the most significant fungal biotic stresses are the rusts that include leaf or brown rust (LR), stem or black rust (SR), and stripe or yellow rust (YR) caused by *Puccinia triticina* Eriks. (*Pt*), *Puccinia graminis* Pers. (*Pgt*), and *Puccinia striiformis* West. (*Pst*), respectively. Among these, LR is the most common rust that is globally distributed and can cause losses from 7 to 30% depending on the developmental stage (Roelfs et al. 1992; Marasas et al. 2004; Bolton et al. 2008; Huerta-Espino et al. 2011). Stem rust occurs mainly in warm weather regions and can cause losses of up to 100% (Leonard and Szabo 2005). Stripe rust occurs in cool, temperate regions, and can cause yield losses ranging from 10 to 70% but up to 100% in highly susceptible cultivars (Chen 2005). The most preferred management strategy for rusts is genetic resistance which is of two types, namely vertical and horizontal (Vanderplank 1963). In a typical vertical resistance, the gene-for-gene interactions between the resistance genes of the host and the avirulence genes of the pathogen form the basis of resistance (Flor 1956). As a result of this incompatible interaction, hypersensitive cell death response is elicited. However, the major problem with this type of qualitative resistance is that it is ephemeral and can be easily overcome by the evolution of new virulent races of the pathogen. For example, the virulent stem rust race group Ug99 carries combined virulence to many genes deployed in the current wheat varieties and poses an enormous threat to global wheat production (Pretorius et al. 2000; Singh et al. 2015). Hence, many breeding efforts focus on horizontal, non-race-specific, quantitative, slow rusting resistance which is the widely preferred mechanism to achieve durability, defined as the ability of a widely deployed resistance gene to provide an economic level of protection over an extended period of time (Johnson 1984). In this type of resistance, although the infection is not completely stopped, the spread of the disease is delayed and it is typically expressed in the adult plant stage (McIntosh et al. 1995). To date, about 76 LR resistance (*Lr*) genes, 59 SR resistance (*Sr*) genes, 76 YR resistance (*Yr*) genes, and several quantitative trait loci (QTL) have been identified (McIntosh et al. 2016). Among these, the known race non-specific resistance genes are *Lr34*/*Yr18*/*Sr57, Lr46*/*Yr29*/*Sr58, Lr67*/*Yr46*/*Sr55, Lr68, Sr2*/*Lr27*/*Yr30*, and *Yr36*.

Breeding for quantitative disease resistance is a challenge because of its complex inheritance, and it is important to devise strategies for more effective evaluation and exploitation of this resistance. With this focus of accelerating breeding for quantitative resistance, one promising approach that can potentially provide accurate predictions of the resistance phenotypes, enabling reduced time to parental selection and leading to increased genetic gain from selection, is genomic selection (GS). Genomic selection uses dense genome-wide markers to obtain the genomic estimated breeding values (BVs) of individuals (Meuwissen et al. 2001). It has been shown to be especially effective for improving quantitative traits, both in simulations (Bernardo and Yu 2007; Toosiet et al. 2010; Wong and Bernardo 2008) and in empirical studies (Crossa et al. 2010, 2014; Heslot et al. 2012; Lorenz et al. 2012; Ornella et al. 2012; Rutkoski et al. 2011, 2012, 2014). It uses a ‘training population’ comprising individuals that have been genotyped and phenotyped for traits of interest to generate BVs that can be used in selecting individuals for intermating in the next cycle of selection prior to phenotypic evaluation.

While some studies comparing prediction models have been reported (Lorenzana and Bernardo 2009; Crossa et al. 2010; Heslot et al. 2012), our objective was to compare three genomic prediction models including genomic best linear unbiased prediction (GBLUP), GBLUP A that was GBLUP with selected loci as fixed effects and reproducing kernel Hilbert spaces-markers (RKHS-M) with least-squares (LS) approach that uses selected loci as fixed effects and models incorporating the pedigree relationship including, RKHS-pedigree (RKHS-P), and RKHS markers and pedigree (RKHS-MP), to determine the BVs for seedling and/or adult plant resistance (APR) to LR, SR and YR. The GBLUP is a whole-genome regression approach that uses the genomic relationship matrix (G-matrix) calculated from markers instead of the pedigree relationship matrix. It has successfully been applied in the prediction of complex traits in humans, plants, and animals (de Los Campos et al. 2013; Habier et al. 2013; VanRaden 2008; Yang et al. 2010). The RKHS semi-parametric approach for genomic prediction was proposed by Gianola (2006) and then by Gianola and van Kaam (2008) who argued that genomic interactions are much more complex than what could be handled by the standard parametric models. Several studies have shown its effectiveness in genomic predictions (Crossa et al. 2010; de los Campos et al. 2010; Perez-Rodriguez et al. 2013). RKHS does not assume linearity and it is expected to capture some non-additive effects well. Since, the genetic architecture of seedling and APR to LR, SR, and YR were different, we evaluated different models to determine which of them are appropriate for a given trait.

## Materials and methods

### Plant materials

For this study, we used the 45th and 46th international bread wheat screening nurseries (IBWSN) comprising 333 and 313 lines, respectively. The IBWSNs are large screening nurseries that were initiated in 1967 and consist of 200–400 advanced lines from CIMMYT’s (Centro Internacional de Mejoramiento de Maíz y Trigo) bread wheat breeding program (van Ginkel and Rajaram 1993). These candidates were previously selected for biotic and abiotic stress resistance, grain yield, and end-use industrial quality characteristics. They are evaluated in multiple trials in Mexico and cooperating locations globally. As such they are ideal for building prediction models as they are expected to have useful and novel genes for disease resistance with considerable variation in their BVs.

### Disease evaluation and phenotypic data

#### Seedling evaluation for leaf rust and stripe rust

Seedling evaluations for LR (45th IBWSN–2010 and 2012; 46th IBWSN–2012) and YR (46th IBWSN–2013) were conducted in CIMMYT’s greenhouses at El Batan, Mexico. Rust inoculum was prepared by suspending freshly collected urediniospores (race MBJ/SP for *Pt* and race Mex96.11 for *Pst*) in light mineral oil, Soltrol (Phillips 66 Co., Bartlesville, OK, USA). The plants were inoculated at the two-leaf stage, placed in a dew chamber overnight, and then transferred to the greenhouse where the minimum, maximum, and average temperatures were 16.1, 30.0, and 20.3 °C. The LR seedling infection types (ITs) were recorded 10 days after inoculation using the 0 to 4 scale described in Roelfs et al. (1992). The responses were linearized to a 0–9 scale (; =0, 0 = 0, 1− = 1, 1 = 2, 1+= 3, 2− = 4, 2 = 5, 2+ = 6, 3− = 7, 3 = 8, 3+ = 9 and 4 = 9). For YR, the seedlings were incubated in a dew chamber at 7 °C in the dark for 48 h and then transferred to the greenhouse. The minimum, maximum, and average greenhouse temperatures were 6.3, 30.9, and 17.3 °C, respectively. YR infection types were recorded 14 days post-inoculation using a 0–9 scale as described by McNeal et al. (1971).

#### Adult plant response evaluation for leaf rust, stem rust, and stripe rust

The 45th IBWSN entries were evaluated for APR to: LR at CIMMYT’s headquarters, El Batan, Mexico during the 2010, 2012, and 2013 crop seasons; SR at Kenya Agricultural and Livestock Research Organization (KALRO), Njoro, Kenya during the 2010 and 2011 main seasons; and YR at Quito, Ecuador during the 2011 crop season and at CIMMYT’s research station, Toluca, Mexico during the 2011 and 2013 crop seasons. Similarly, the 46th IBWSN entries were evaluated for APR to LR at El Batan during the 2011 and 2013 crop seasons; SR at KALRO, Njoro during the 2011 main and off seasons; and YR at Toluca during the 2011 and 2013 crop seasons; Quito, Ecuador during the 2012 crop season and KALRO, Njoro during the 2011 main season. The modified Cobb Scale (Peterson et al. 1948) was used to score rust severity at the adult plant stage to determine the percentage of infected tissue (0–100%). Evaluations were conducted at three time points between early and late dough stages. The first evaluation was done when the severity of susceptible check (Avocet) reached 80% followed by two more evaluations at weekly intervals. For all the rust evaluations, the lines were sown in 0.7-m-long-paired rows on top of 30-cm-wide raised beds. For LR, a mixture of the susceptible genotypes ‘Avocet + *Yr24*’ and ‘Avocet + *Yr26*’ was planted as spreader rows around the experimental field. The spreader rows and hills were artificially inoculated with urediniospores of the two prevalent Mexican *Pt* races, MBJ/SP and MCJ/SP suspended in Soltrol oil to initiate an epidemic. These two races differ by their virulence to the *Lr26* gene (MBJ/SP has partial virulence for *Lr26*, while MCJ/SP has complete virulence). The inoculations were carried out twice when the plants were at the 6-leaf stage. For SR evaluation, a border row of spreaders was planted surrounding the field and sprayed twice with fresh urediniospores of *Pgt* race TTKST suspended in Soltrol to create an artificial rust epidemic. The plants within the border rows were inoculated by injecting a suspension of freshly collected urediniospores in water using a hypodermic syringe, twice prior to booting (growth stage Z35-Z37) (Zadoks et al. 1974). For YR evaluation, spreaders consisted of a mixture of six susceptible wheat lines derived from an Avocet/Attila cross. The 4-week-old spreaders and hills were inoculated three times, at 3–4 day intervals with mixed *Pst* isolates, Mex96.11, and Mex08.13. While Mex96.11 is virulent to *Yr27* and avirulent to *Yr31*, it is the reverse for Mex08.13. There were replicated controls/local checks every 20 lines for all the evaluations.

The phenotypic data for all the diseases were transformed to normal distributions by identifying appropriate exponent (lambda) values using the boxcox (Box and Cox 1964) function in the ‘R’ statistical program.

### Genotyping

The nurseries were genotyped using the genotyping-by-sequencing (GBS) method to obtain dense genome-wide coverage (Elshire et al. 2011). GBS markers were obtained using the method described by Poland et al. (2012). After filtering for markers with missing data greater than 50%, minor allele frequency less than 10%, and pairwise marker correlation (*r* ^2^) greater than 0.95 (for redundancy), 5102 markers for the 45th IBWSN and 8066 markers for the 46th IBWSN were obtained. Missing data were imputed using the expectation–maximization algorithm implemented in the ‘R’ package rrBLUP (Endelman 2011). The lines were also filtered for missing data greater than 50% which resulted in 267 and 305 lines in the 45th and 46th IBWSN, respectively.

### Relationship matrix and heritability estimation

The G-matrix was calculated according to VanRaden (2008) and implemented in the ‘R’ package rrBLUP (Endelman 2011). The relationship matrix was centered and standardized for all the analyses. Heritability was calculated on a line-mean basis and estimates of the genetic and residual variances were obtained using the average information-restricted maximum likelihood algorithm (Gilmour et al. 1995) implemented in the ‘heritability’ package in ‘R’ (Kruijer et al. 2015).

### Prediction models

#### Least squares (LS)

A stepwise least-squares (LS) approach was used which involves an initial marker ranking and selection step. First, genome-wide association analysis was conducted in the training set to calculate marker *p* values. Then, the markers were ranked according to their *p* values for variable selection. For each iteration* i* through* j*, a marker was added to the model, starting from the marker with the lowest *p* value,

$$y={1_n}\mu +{X_i}{\beta _i} \ldots \ldots {X_j}{\beta _j}+\varepsilon$$

where *y* is the phenotype, *µ* is the mean, *β*_i_ denotes the effect of the *i*th marker, and *X*_*i*_ denotes the *i*th marker’s genotype matrix. The fivefold cross-validation accuracy was calculated within the training set after each iteration and the model with *j* − 1 markers was selected when the Accuracy_*j*−1_>Accuracy_*j*_. The second step involved marker effects estimation from the selected model that was then used to predict the BVs of the individuals. To obtain the chromosomal locations of the significant markers, the basic local alignment search tool (BLAST) (https://triticeaetoolbox.org/wheat/viroblast/viroblast.php) in the Triticeae Toolbox website was used. A nucleotide BLAST (BLAST-n) was performed against the wheat markers in Triticeae Toolbox (T3) database (updated on April 2015), wheat contigs (1A to 7D) from the wheat CSS genome reference v2, September 2014, wheat chromosomes (1A to 7D) and unsorted scaffolds from IWGSC1.0 + popseq (November 2014) (Chapman et al. 2015). This approach would help to identify markers that are similar in other populations genotyped by GBS and also enable us to compare across studies using marker synonyms.

### Genomic best linear unbiased prediction (GBLUP) and GBLUP with selected loci as fixed effects (GBLUP A)

For GBLUP, the BVs of individuals were predicted using the mixed model:

$$ {\mathbf{y}}=1_n {\varvec{\mu}} + {\user2{Zu}} + \varvec {\varepsilon} $$

where ***y*** is the vector of the response phenotypic trait, ***µ*** is the overall mean vector, ***u*** is the vector of genotype effects that are assumed to be multivariate normal random effects [***u***~*N*(**0, G**σ^2^_u_), ***Z*** is the design matrix for the random effects, and **ε** is the vector of independent residuals assumed to have a multivariate normal distribution (***ε***~*N*(**0, I**σ^2^_e_)]. The ‘R’ package, rrBLUP (Endelman 2011) was used to implement GBLUP. We also evaluated GBLUP A model, that, in addition to the GBLUP model, also included some loci modeled as fixed effects, selected by the same method described for the LS model:

$$y={1_n}\mu +{X_i}{\beta _i} \ldots \ldots {X_j}{\beta _j}+Zu+\varepsilon .$$

### Reproducing kernel Hilbert spaces (RKHS)

The RKHS model using a Gaussian kernel is of the form:

$${y_i}={{\bf w}_i}^\prime \beta +{{\bf z}_i}^\prime {\bf u}+\;\sum\limits_{j=1}^n {\exp \left[ {\frac{{ - ({x_i} - {x_j})'\;({x_i} - {x_j})}}{h}} \right]{\alpha _j}+\varepsilon_i}$$

where *x*_*i*_ and *x*_*j*_ are the observed marker genotypes of individuals, ***w***_*i*_ and ***Z***_*i*_ are the incidence vectors, ***β*** is the vector of location effects, ***u*** is the vector of additive genetic effects, ${\alpha _j}$ is the regression coefficient, and **ε**_**i**_ is the error term [***ε***_*i*_–*N*(0, **I**σ^2^_e_)] (Gianola 2006). The additive genetic effects ***u***~N(0, ***K***σ^2^_g_), where ***K*** is the reproducing Gaussian kernel, $K(x_i,x_j)=\exp \left[ {\frac{{({x_i} - {x_j})'({x_i} - {x_j})}}{h}} \right]$ and *h* is the bandwidth parameter. We implemented three RKHS models in the BGLR package (Perez and de los Campos 2014), namely (1) RKHS markers (RKHS-M) using the G-matrix calculated from markers, (2) RKHS-pedigree (RKHS-P) using the pedigree relationship matrix which was obtained from the pedigree and was twice the coefficient of ancestry, and (3) RKHS markers and pedigree (RKHS-MP) with the marker and pedigree relationship matrices as two kernels, where the additive effect was captured by regression on the markers and also with the (co)variance relationship derived from the pedigree. We fitted these models with three arbitrarily chosen bandwidth parameters and then averaged the three accuracies.

### Prediction accuracies

The predictive ability of the models was assessed using the Pearson’s correlation between the observed and the cross-validated estimated BVs, which is the prediction accuracy. We used the tenfold cross-validation where the whole dataset was divided into tenfolds and nine of them (240 lines and 275 lines in the 45th and 46th IBWSN, respectively) were used as a training set to estimate the marker effects, which were then used to predict the BVs in the 10th fold, referred to as the validation set (27 lines and 30 lines in the 45th and 46th IBWSN, respectively).

## Results

### Phenotypic data analysis

**Fig. 1:**
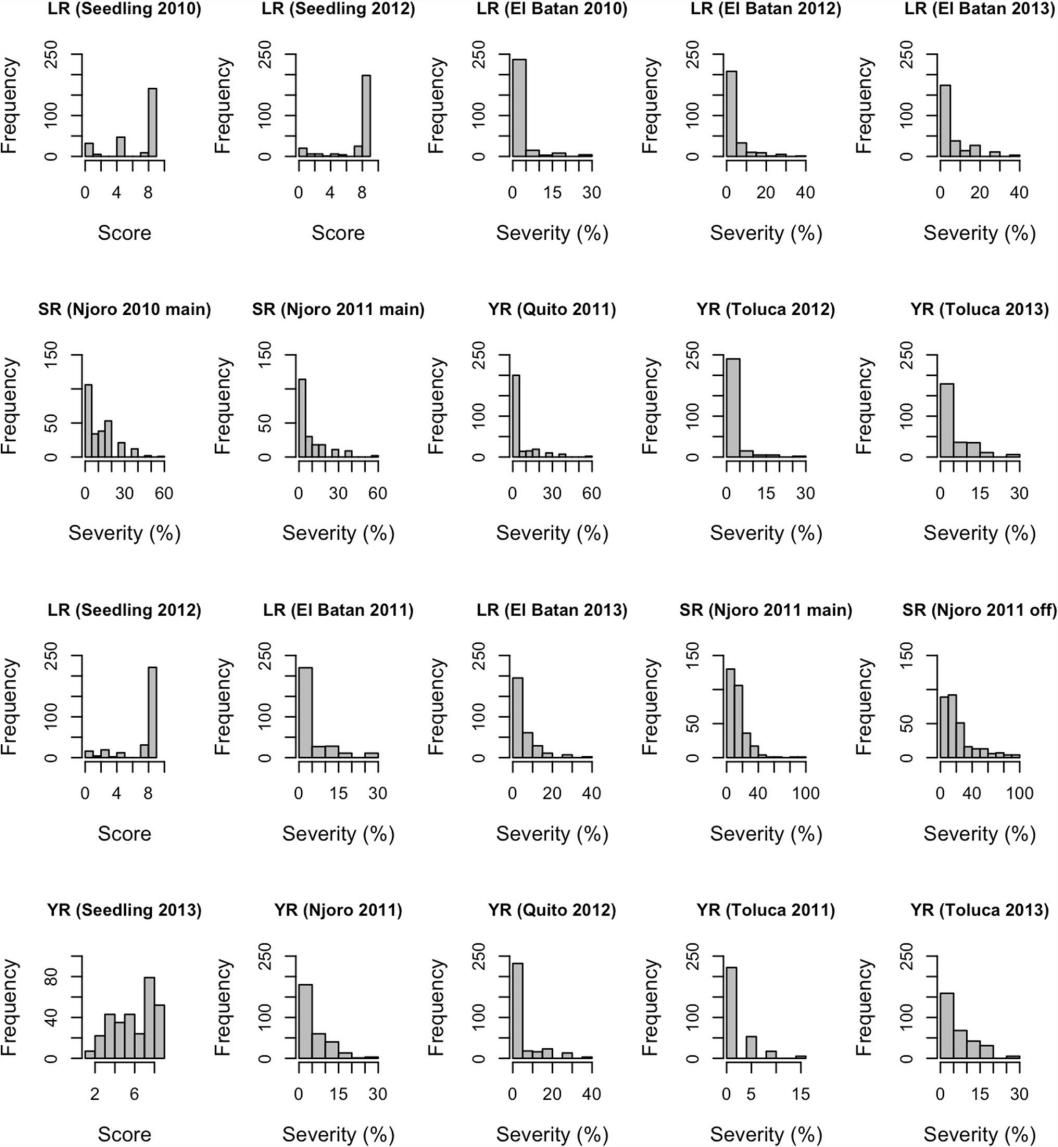
Phenotypic distributions for leaf rust (LR), stem rust (SR), and stripe rust (YR) in the 45th (*top two panels*) and 46th (*lower* *two panels*) international bread wheat screening nurseries (IBWSN)

The phenotypic distributions of the rusts in the 45th and 46th IBWSN are shown in Fig. 1. In both nurseries, the average correlation between LR seedling resistance and APR was very low (0.1 and 0.3 for the 45th and 46th IBWSN, respectively) indicating that the genetic bases of seedling resistance and APR were different.

### Relationship and heritability analysis

**Fig. 2:**
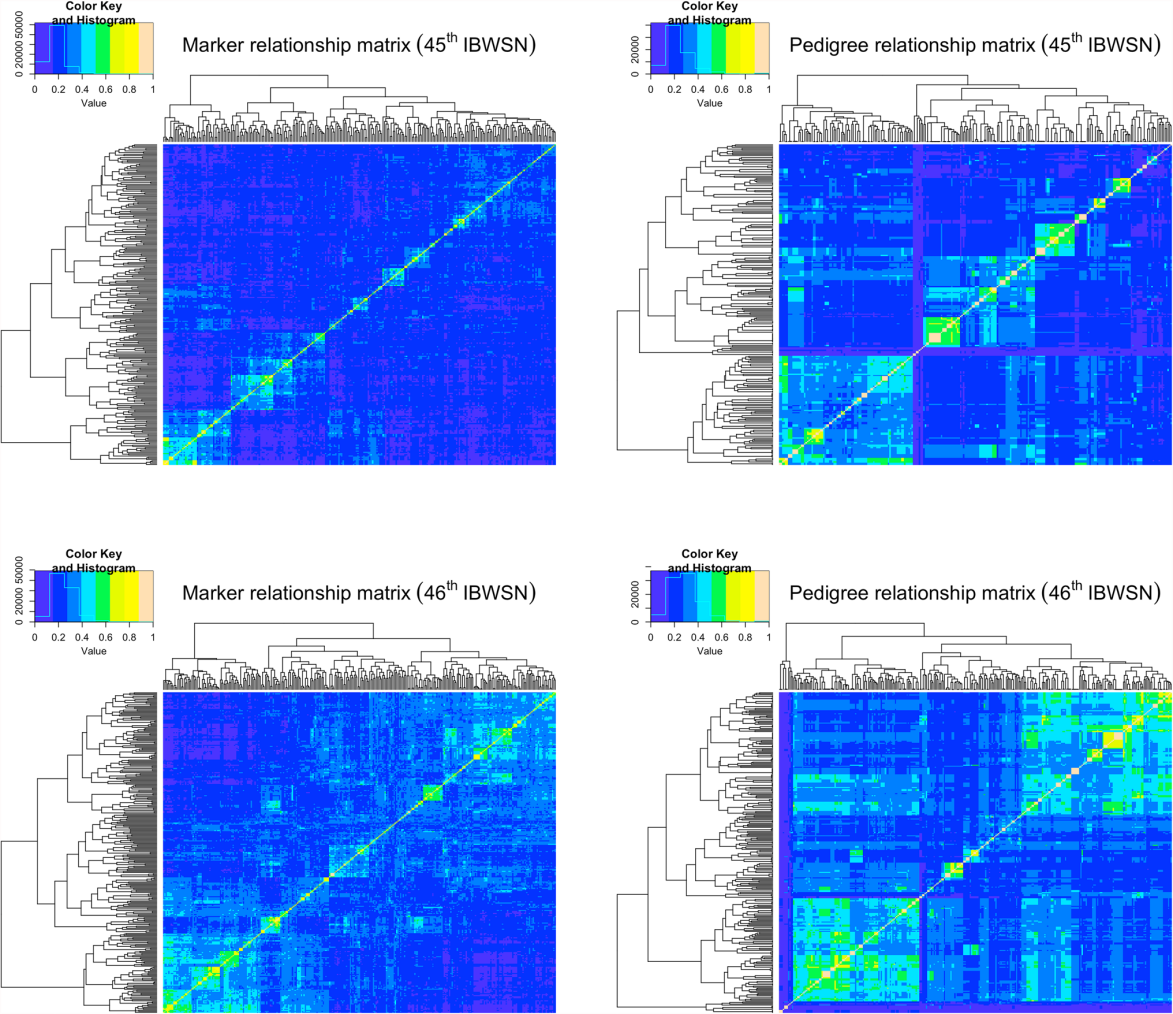
Heat map of the marker and pedigree-based relationship matrices for the 45th and 46th international bread wheat screening nurseries (IBWSN) illustrating the familial relatedness (kinship) between the individuals

Heatmap of the genomic and the pedigree-based relationship matrices for the 45th and 46th IBWSN (Fig. 2) indicated that the lines in the 46th IBWSN had a slightly higher relationship among them than those in the 45th IBWSN. The 267 lines in the 45th IBWSN comprised one family with eight full-sibs, one with six full-sibs, one with five full-sibs, seven with four full-sibs, 15 with three full-sibs, 37 with two full-sibs, and 101 crosses represented by one individual per cross. The 305 lines in the 46th IBWSN comprised one family with seven full-sibs, two with six full-sibs, seven with four full-sibs, 12 with three full-sibs, 34 with two full-sibs, and 154 with one individual per cross. We also observed that the pedigree relationship matrices for both nurseries indicated a higher relationship among the lines than the marker-based matrices, because it does not account for Mendelian sampling. In the 45th IBWSN, the broad-sense line-mean heritability was the highest for LR seedling (0.72) followed by SR APR (0.59), LR APR (0.58), and YR APR (0.26). In the 46th IBWSN, the highest heritability was obtained for LR APR (0.6), followed by SR APR (0.5) and YR APR (0.48). The broad-sense heritability was very high for LR seedling (0.87) and YR seedling (0.86).

### Markers significantly associated with leaf, stem, and stripe rust resistance

The markers that were significantly associated with LR, SR, and YR resistance in the 45th and 46th IBWSN and used as fixed effects in the LS model are shown in Tables 1 and 2, respectively. Only the markers that perfectly matched with a marker in the T3 database and were significant in at least fivefolds are reported. The BLAST results for all the markers and other synonyms are reported in Supplementary Table 1. For LR seedling resistance in the 45th IBWSN, marker GBS_24751 (0 cM) on chromosome 2BS explained the highest variation (18%) in the 2010 dataset. Marker GBS_37247 on chromosome 1DS was significant in both the replications and explained 15 and 24% of the average variation. In the 46th IBWSN, marker GBS_19971 on chromosome 1DS was significant in all the folds and explained an average of 33% of the variation for LR seedling resistance. In addition, a marker on chromosome 3B and another marker on chromosome 1DS were also significant. For LR APR in the 45th IBWSN, the marker GBS_30281 on chromosome 4AL was significant in all the three datasets and explained 8–12% of the average variation. The only other significant marker with known position was GBS_8842 on chromosome 3AS in the 2010 dataset. In the 46th IBWSN, GBS_40747 on chromosome 2D was significantly associated with LR APR in the El Batan 2011 dataset and explained 10% of the average variation. In addition, markers GBS_18425 and GBS_2400 both on chromosome 3AS and GBS_1491 on chromosome 3AL were significant in the El Batan 2013 dataset.

**Table 1:** Quantitative trait loci (QTL) linked markers used as fixed effects in the least-squares (LS) model for the 45th international bread wheat screening nursery (IBWSN)

Dataset	Marker	Marker synonym^a^	Chromosome	Genetic position in cM (Popseq map)	Physical position in bps (Popseq map)	Expected genes	Average *p* value	Average *R* ^2^	Frequency^b^
Leaf rust									
Seedling 2010	GBS_24751	gbsHWWAMP38350	2BS	0	3,863,480	*Lr16*	2.15E−10	0.18	0.8
	GBS_37247	WCSS1_contig470290_1DS-434	1DS	2.7	1,241,625	*Lr42*	2.57E−09	0.15	0.5
Seedling 2012	GBS_37247	WCSS1_contig470290_1DS-434	1DS	2.7	1,241,625	*Lr42*	4.33E−14	0.24	1
	GBS_38357	–	–	–	–	–	2.04E−10	0.17	0.8
El Batan 2010	GBS_8842	WCSS1_contig3334901_3AS-4592	3AS	9.4	3,823,808	–	5.24E−07	0.12	1
	GBS_30281	WCSS1_contig7120458_4AL-2167	4AL	121.9	210,542,445	–	2.13E−07	0.12	0.9
El Batan 2012	GBS_12317	–	–	–		–	3.42E−06	0.09	0.5
	GBS_30281	WCSS1_contig7120458_4AL-2167	4AL	121.9	210,542,445	–	6.67E−06	0.08	0.5
El Batan 2013	GBS_1926	–	–	–	–	–	5.60E−08	0.12	0.9
	GBS_30281	WCSS1_contig7120458_4AL-2167	4AL	121.9	210,542,445	–	6.21E−08	0.12	0.9
	GBS_5135	–	–	–	–	–	2.69E−07	0.11	0.5
Stem rust									
Njoro 2010	GBS_22856	WCSS1_contig10511286_3B-5360	3B	–	90,941,978	*Sr2*	3.89E−10	0.16	1
	GBS_36529	WCSS1_contig10759567_3B-1965	3B	76.4	370,963,380	*Sr12*	9.17E−09	0.14	0.5
	GBS_2454	WCSS1_contig2284473_5BS-12,686	5BS	4.2	4,471,954	–	3.19E−09	0.14	0.5
Njoro 2011	GBS_22856	WCSS1_contig10511286_3B-5360	3B	–	90,941,978	*Sr2*	1.04E−08	0.18	1
	GBS_13047	gbsHWWAMP18106	3B	0	312,463	*Sr2*	8.10E−08	0.17	0.9
	GBS_23598	–	–	–	–		1.20E−07	0.15	0.7
Stripe rust									
Quito 2011	GBS_6432	WCSS1_contig5219749_2AS-4945	2AS	8.8	6,792,755	*Yr17*	2.91E−10	0.16	1
Toluca 2012							2.41E−13	0.21	1
Toluca 2013	GBS_702	WCSS1_contig5304580_2AS-10,182	2AS	0	944,474		0.0E+00	0.29	1
	GBS_6432	WCSS1_contig5219749_2AS-4945	2AS	8.8	6,792,755		0.0E+00	0.32	0.9

^a^Markers prefixed by gbsHWWAMP are from the hard winter wheat association mapping panel available in T3 database and markers prefixed by WCSS1_contig are from the CSS GBS 2014 physical map, where ‘WCSS1’ stands for wheat chromosome survey sequence

^b^The frequency of the marker in the ten cross-validation folds

**Table 2:** Quantitative trait loci (QTL) linked markers used as fixed effects in the least-squares (LS) model for the 46th international bread wheat screening nursery (IBWSN)

Dataset	Marker	Marker synonym^a^	Chromosome	Genetic position in cM (Popseq map)	Physical position in bps (Popseq map)	Expected genes	Average *p* value	Average *R* ^2^	Frequency^b^
Leaf rust									
Seedling 2012	GBS_19971	WCSS1_contig1905752_1DS-4628	1DS	5.4	2,073,708	*Lr42*	<E−16	0.33	1
	GBS_28186	gbsHWWAMP44231	3B	25.3	15,366,258	–	<E−16	0.26	1
	GBS_28376	WCSS1_contig1898017_1DS-2235	1DS	11.0	3,306,922	*Lr42*	<E−16	0.39	0.9
El Batan 2011	GBS_40747	gbsHWWAMP55344	2D	17.3	8,198,944	–	3.08E−07	0.10	0.8
	GBS_38496	–	–	–	–	–	2.27E−07	0.10	0.7
El Batan 2013	GBS_18425	WCSS1_contig3419689_3AS-1090	3AS	60.6	61,387,121	–	5.08E−10	0.14	1
	GBS_2400	WCSS1_contig3361063_3AS-2705	3AS	53.4	14,901,099	–	2.83E−10	0.13	0.6
	GBS_1491	gbsHWWAMP1393	3AL	63.1	133,091,112	-	3.70E−10	0.14	0.5
Stem rust									
Njoro 2011 main	GBS_23856	gbsHWWAMP37196	1AL	86.5	220,028,370	–	3.44E−08	0.12	0.9
	GBS_1505	–	3B	–	91,939	*Sr2*	6.79E−08	0.11	0.5
Njoro 2011 off	GBS_28025	WCSS1_contig3042477_6BS-5453	6BS	65.1	70,672,093	–	1.18E−08	0.13	1
	GBS_1505	–	3B	–	91,939	*Sr2*	1.03E−07	0.11	0.6
	GBS_20060	WCSS1_contig2078323_6DS-22,086	6DS	2.5	2,067,639	*SrTmp*	1.33E−08	0.12	0.5
Stripe rust									
Seedling 2013	GBS_702	WCSS1_contig5304580_2AS-10,182	2AS	0	944,474	*Yr17*	<E−16	0.58	1
Quito 2012							<E−16	0.26	1
Njoro 2011							<E−16	0.27	1
Toluca 2011							<E−16	0.41	1
Toluca 2013							<E−16	0.41	1

^a^Markers prefixed by gbsHWWAMP are from the hard winter wheat association mapping panel available in T3  database and markers prefixed by WCSS1_contig are from the CSS GBS 2014 physical map, where ‘WCSS1’ stands for wheat chromosome survey sequence

^b^The frequency of the marker in the ten cross-validation folds

For SR APR, in the 45th IBWSN, the marker GBS_22856 on chromosome 3B was significantly associated and explained an average variation of 16 and 18% in the Njoro 2010 and 2011 datasets, respectively. In addition, markers GBS_36529 on chromosome 3B and GBS_2454 on chromosome 5B were significant in the Njoro 2010 dataset and marker GBS_13047 on chromosome 3B was significant in the Njoro 2011 dataset. In the 46th IBWSN, GBS_23856 on chromosome 1AL and GBS_1505 on chromosome 3B were significant in the Njoro 2011 main season. Markers, GBS_28025 on chromosome 6BS, GBS_1505 on chromosome 3B, and GBS_20060 on chromosome 6DS, were significant in the 2011 off season. For YR seedling (2013) in the 46th IBWSN, the marker GBS_702 on chromosome 2AS was significant in all the folds and explained an average 58.5% of the variation. For YR APR, the marker GBS_6432 on chromosome 2AS was significant in all the datasets and explained an average of 16 to 32% of the variation. GBS_702 on chromosome 2AS was significant in all the folds in the Toluca 2013 dataset and explained 29% of the average variation. In the 46th IBWSN, the marker, GBS_702 on chromosome 2AS, was significant in all the folds in all the YR APR datasets and explained an average variation of 26 to 41%.

### Prediction accuracies

Prediction accuracies for LR, SR, and YR resistance in the 45th and 46th IBWSNs are shown in Table 3.

**Table 3:** Prediction accuracies for leaf rust (LR), stem rust (SR), and stripe rust (YR) resistance in the 45th and 46th international bread wheat screening nurseries (IBWSN)

Trait	Dataset	IBWSN	LS	GBLUP	GBLUP A	RKHS-M	RKHS-P	RKHS-MP
Leaf rust	Seedling 2010	45th	0.31 ± 0.09	0.7 ± 0.03	0.69 ± 0.03	0.71 ± 0.03	0.7 ± 0.03	0.74 ± 0.03
	Seedling 2012	45th	0.42 ± 0.1	0.58 ± 0.05	0.6 ± 0.05	0.59 ± 0.05	0.73 ± 0.05	0.72 ± 0.05
	Seedling 2012	46th	0.66 ± 0.04	0.64 ± 0.05	0.7 ± 0.03	0.65 ± 0.05	0.61 ± 0.07	0.67 ± 0.05
	El Batan 2010	45th	0.34 ± 0.05	0.43 ± 0.05	0.43 ± 0.04	0.43 ± 0.05	0.42 ± 0.07	0.46 ± 0.05
	El Batan 2012	45th	0.12 ± 0.07	0.41 ± 0.05	0.26 ± 0.07	0.41 ± 0.05	0.34 ± 0.06	0.41 ± 0.05
	El Batan 2013	45th	0.29 ± 0.06	0.47 ± 0.06	0.44 ± 0.06	0.48 ± 0.06	0.5 ± 0.06	0.52 ± 0.06
	El Batan 2011	46th	0.28 ± 0.05	0.51 ± 0.04	0.49 ± 0.05	0.51 ± 0.04	0.5 ± 0.03	0.53 ± 0.04
	El Batan 2013	46th	0.38 ± 0.03	0.52 ± 0.04	0.51 ± 0.03	0.53 ± 0.03	0.48 ± 0.03	0.56 ± 0.03
Stem rust	Njoro 2010 main	45th	0.41 ± 0.05	0.59 ± 0.04	0.64 ± 0.03	0.59 ± 0.04	0.63 ± 0.03	0.65 ± 0.03
	Njoro 2011 main	45th	0.41 ± 0.08	0.59 ± 0.05	0.62 ± 0.04	0.59 ± 0.05	0.53 ± 0.07	0.58 ± 0.06
	Njoro 2011 main	46th	0.31 ± 0.04	0.54 ± 0.05	0.54 ± 0.05	0.54 ± 0.05	0.55 ± 0.06	0.62 ± 0.05
	Njoro 2011 off	46th	0.31 ± 0.03	0.47 ± 0.06	0.43 ± 0.05	0.47 ± 0.06	0.34 ± 0.04	0.45 ± 0.06
Stripe rust	Seedling 2013	46th	0.77 ± 0.03	0.73 ± 0.03	0.78 ± 0.02	0.73 ± 0.03	0.7 ± 0.03	0.74 ± 0.03
	Quito 2011	45th	0.37 ± 0.05	0.39 ± 0.06	0.41 ± 0.06	0.38 ± 0.07	0.34 ± 0.08	0.39 ± 0.07
	Toluca 2012	45th	0.45 ± 0.04	0.39 ± 0.04	0.45 ± 0.04	0.39 ± 0.05	0.37 ± 0.04	0.39 ± 0.04
	Toluca 2013	45th	0.55 ± 0.03	0.69 ± 0.02	0.7 ± 0.02	0.68 ± 0.02	0.66 ± 0.03	0.7 ± 0.03
	Quito 2012	46th	0.51 ± 0.03	0.55 ± 0.03	0.6 ± 0.03	0.54 ± 0.03	0.58 ± 0.03	0.61 ± 0.03
	Njoro 2011	46th	0.51 ± 0.03	0.52 ± 0.03	0.56 ± 0.03	0.52 ± 0.04	0.55 ± 0.04	0.56 ± 0.04
	Toluca 2011	46th	0.63 ± 0.03	0.6 ± 0.03	0.65 ± 0.03	0.59 ± 0.02	0.64 ± 0.02	0.63 ± 0.02
	Toluca 2013	46th	0.63 ± 0.04	0.68 ± 0.03	0.71 ± 0.04	0.68 ± 0.03	0.55 ± 0.06	0.66 ± 0.04

*IBWSN* International bread wheat screening nursery, *LS* least squares, *GBLUP* genomic best linear unbiased prediction, *GBLUP A* genomic-BLUP with selected loci as fixed effects, *RKHS-M* reproducing kernel Hilbert spaces-markers, *RKHS-P* reproducing kernel Hilbert spaces pedigree, *RKHS-MP* reproducing kernel Hilbert spaces-markers and pedigree

### Prediction accuracies for leaf rust seedling and adult plant resistance

For LR seedling in the 45th IBWSN, the highest prediction accuracy was obtained using the RKHS-MP and RKHS-P, respectively in the 2010 and 2012 datasets. The lowest accuracy was obtained using the LS approach and GBLUP resulted in 125.8 and 38.1% increase in accuracy over LS in the two datasets. While RKHS-P model performed similar to the other genome-wide models in the 2010 dataset, it gave a 23.7% increase in accuracy over the RKHS-M in the 2012 dataset. There were no significant differences in the accuracies obtained from GBLUP, GBLUP A, and RKHS-M. In the 46th IBWSN, the highest accuracy for LR seedling resistance was obtained using GBLUP A followed by RKHS-MP, LS, RKHS-M, and GBLUP which gave similar accuracies. The RKHS-P model yielded the lowest prediction accuracy, but it was only 6.55% lower than RKHS-M. For LR APR, it was observed that RKHS-MP gave the highest accuracies and LS, the lowest in all five datasets. The increase in accuracy obtained from using GBLUP over LS varied across the different datasets and ranged from 26.5 to 241.7%. GBLUP A performed similar to GBLUP in all the datasets except in the El Batan 2012 dataset (45th IBWSN), where the fixed effect markers explained very little variation. The accuracies obtained using pedigree and genome-wide marker-based models were not significantly different in all the datasets, but there was a slight increase in accuracy using genome-wide markers in the El Batan 2012 (45th IBWSN) and El Batan 2013 (46th IBWSN) datasets (20.6 and 10.4% respectively). GBLUP and RKHS-M gave similar accuracies in all the datasets.

### Prediction accuracies for stem rust adult plant resistance

For SR APR, the lowest prediction accuracy in all four datasets was obtained using LS and GBLUP resulted in 43.9–74.2% increase in accuracy over LS. The highest accuracy was obtained with RKHS-MP in two datasets, GBLUP A in one dataset and with both GBLUP and RKHS-M in the other dataset. The RKHS-P model performed similar to GBLUP in one dataset and slightly better than GBLUP (6.8% increase in accuracy) in another dataset. However, we observed a decrease in accuracy of 10.2 and 27.7% using the RKHS-P vs RKHS-M in two datasets. As observed for LR, GBLUP and RKHS-M gave similar accuracies in all the datasets.

### Prediction accuracies for stripe rust seedling and adult plant resistance

For YR seedling resistance in the 46th IBWSN, the highest accuracies were obtained using GBLUP A followed by LS, RKHS-MP, GBLUP, RKHS-M, and RKHS-P models. Although RKHS-P gave the lowest accuracy, the increase in accuracy using RKHS-M over the pedigree was only 4.3%. Least squares performed slightly better than GBLUP and resulted in 5.5% increase in accuracy. For YR APR, in the 45th IBWSN, the highest accuracy was obtained with GBLUP A in the Quito 2011 dataset, with LS and GBLUP A in the Toluca 2012 dataset and with GBLUP A and RKHS-MP in the Toluca 2013 dataset. Least squares performed similar to the GBLUP in the Quito 2011 dataset, slightly better than GBLUP in the Toluca 2012 dataset (15.4% increase in accuracy) and poorer than GBLUP (20.3% decrease in accuracy) in the Toluca 2013 dataset. Although the RKHS-P model gave the lowest accuracies in two datasets, the increase in accuracy using markers was not significant (ranged from 3 to 11.8%). The GBLUP, RKHS-M, and RKHS-MP models gave similar accuracies in all the datasets. In the 46th IBWSN, RKHS-MP gave the highest accuracy in the Quito 2012 dataset; RKHS-MP and GBLUP A in the Njoro 2011 dataset; and GBLUP A in the Toluca 2011 and 2013 datasets. The RKHS-P model performed similar to RKHS-M in all the datasets, except the Toluca 2013 dataset where RKHS-M resulted in 23.6% increase in accuracy over RKHS-P. We also observed that LS performed similar to GBLUP in the Njoro 2011 dataset, slightly better than GBLUP in the Toluca 2011 dataset (5% increase in accuracy) and slightly poorer than GBLUP in the Quito 2012 and Toluca 2013 datasets (7.3 and 7.4% decrease in accuracy). GBLUP and RKHS-M models yielded similar accuracies in all the datasets.

## Discussion

Genomic prediction for LR seedling resistance resulted in 82% average increase in accuracy over LS in the 45th IBWSN. However, LS performed similar to genome-wide marker models in the 46th IBWSN. This can be attributed to the two significant markers on chromosome 1DS (5.4 and 11 cM) that were used as fixed effects and explained a large percent of the variability in the folds (33% and 39%). In this case, genome-wide markers would not be required for high accuracy, suggesting that the genetic architecture of resistance in a given population is an important factor that determines the appropriate model. We believe these markers to be linked to the *Lr42* seedling resistance gene based on their distal location in the chromosome and also the presence of this gene in Quaiu (Basnet et al. 2013), which was used as a parent for several crosses. Simple sequence repeat markers, *cfd15* and *wmc432*, also confirmed the presence of the *Lr42* gene in some lines from these nurseries. A marker at about the same position on chromosome 1DS (2.7 cM) was also significant in both the datasets in the 45th IBWSN. While it could also be linked to the *Lr42* gene, it explained only 15 to 24% of the variability in this nursery and resulted in lower accuracies using the LS. There was also another marker at the distal end of chromosome 2BS (0 cM) used as a fixed effect in the 2010 dataset, which is likely to be linked to *Lr16*, a seedling effective race-specific resistance gene. *Lr16* is present at a high frequency in CIMMYT germplasm, especially in lines derived from Waxwing and Francolin parentage (Lan et al. 2014), which were used as parents in several crosses.

Genomic prediction for APR to LR and SR yielded an average increase in accuracy of 89.8% and 53.4% respectively, over LS in both the nurseries. Because LR and SR APR had moderate heritabilities and are quantitative traits conditioned by many genes with small effects, the poor performance of LS was expected. For LR APR in the 45th IBWSN, the marker on chromosome 4AL that was significant in all the datasets did not coincide with any of the known genes which are effective to this *Pt* race and may be identifying a novel QTL. A marker on chromosome 3AS (9.4 cM) was significant in all the folds in the 2010 dataset. Although *Lr63* is the only known gene mapped to the distal end of chromosome 3AS (Kolmer et al. 2010), it is unlikely that it is present in these lines considering its origin. In the 46th IBWSN, a marker on chromosome 2D (17.3 cM) was significant in the 2011 dataset and three markers on chromosome 3A (53.4–63.1 cM) were significant in the 2013 dataset. Since their positions could not be compared to any of the known genes in these chromosomes and the catalogued genes are not effective to this *Pt* race, they might be identifying novel QTL. Stem rust APR in the 45th IBWSN was associated with markers at two locations on chromosome 3B (0 and 76.4 cM). The marker at the distal end of chromosome 3B might be linked to the durable stem rust resistance gene, *Sr2* which is present in a high frequency in CIMMYT lines. The other marker on chromosome 3B might be linked to the *Sr12* resistance gene, which despite being ineffective against Ug99 alone, was suggested to confer APR in combination with other resistance loci by complementary epistasis (Rouse et al. 2014). *XwPt6047*, the marker closely linked to the *Sr12* gene (Rouse et al. 2014) is located at 52.7 cM in the CIMMYT integrated DArT map (Crossa et al. 2007), but it was not possible to obtain its relative position in the popseq map. In addition to the markers on chromosome 3B, a marker at the distal end of chromosome 5BS (4.2 cM) was also significant. While it is was not possible to determine what gene it was linked to, a minor QTL for Ug99 resistance has been reported on the distal end of chromosome 5BS by Yu et al. (2011). In the 46th IBWSN, SR APR was associated with a marker on an unknown location on chromosome 3B in both seasons and one marker each on chromosome 1AL (86.5 cM), chromosome 6BS (65.1 cM), and chromosome 6DS (2.5 cM). The position of the markers on chromosomes 1AL and 6BS could not be compared to previously reported Ug99 resistance QTL as relative markers were not available. We believe that the marker on chromosome 6DS is linked to the *SrTmp* gene, but it is no longer effective against Ug99 (Newcomb et al. 2016).

For seedling resistance to YR, we observed that GBLUP A and LS performed slightly better than GBLUP. This can be attributed to the very high heritability of the trait and the marker, GBS_702 on chromosome 2AS that explained a large variation in the folds. This is another case where genomic prediction is not necessary for high accuracy. For YR APR in the 45th IBWSN, GBLUP A performed the best in all the datasets and LS also performed well except in one dataset. This is due to markers GBS_6432 and GBS_702 on chromosome 2AS that explained a large variation. Similarly, in the 46th IBWSN, the GBLUP A model had the highest accuracy in most datasets and the high accuracies obtained from both LS and GBLUP A were due to the marker, GBS_702 on chromosome 2AS that had a large effect. Unlike LR and SR, APR to YR in these nurseries behaved as a simple trait and could be predicted well using LS. The significant association of the same marker to both seedling resistance and APR indicates that it is an all-stage resistance gene that we believe to be *Yr17* or a closely linked gene. The *Yr17* gene is located at the distal end of chromosome 2AS which is also the location of GBS_702 (0 cM) and GBS_6432 (8.8 cM). Many lines with Kachu, Milan, and Mutus in the pedigree are expected to have the *Yr17* gene. *Yr17* was introgressed into the French wheat cultivar, VPM-1 as a translocation segment from the D-genome of *Aegilops ventricosa*. The sequence-tagged site marker, *Ventriup* + *Ln2* and the single nucleotide polymorphism marker, *CIMwMAS0004* that amplify a region in this translocation confirmed its presence in about 45% of the lines in the 45th and 46th IBWSN, respectively. Although *Yr17* is closely linked to *Lr37* and *Sr38*, it is to be noted that races MBJ/SP and MCJ/SP are virulent to *Lr37* and the Ug99 group of races in Kenya are virulent to *Sr38*.

Overall, our prediction results indicate that genome-wide marker-based prediction models were more accurate than LS in most datasets, which is consistent with several previous studies (Meuwissen et al. 2001; Bernardo and Yu 2007; Habier et al. 2007; Muir 2007; Piyasatian et al. 2007; Lorenzana and Bernardo 2009; Moser et al. 2009; Heffner et al. 2011a, b; Rutkoski et al. 2012, 2014). Only a few markers were included in the LS model for some traits, because the other markers explained a very small portion of the variance and did not improve the predictions. We obtained an average of 42% increase in accuracy using the GBLUP compared to LS. This is comparable to the previous reports: Meuwissen et al. (2001) obtained a 41% greater accuracy using RR-BLUP than stepwise regression in simulations; Bernardo and Yu (2007) obtained an 18 and 43% improvement in the responses using GS compared to marker-assisted recurrent selection in their simulation study for a trait that has high and low heritability; Piyasatian et al. (2007) obtained a 32% increase in accuracy using RR over stepwise regression in earlier generations; Heffner et al. (2011a) reported 28% higher average accuracies using GS than marker-assisted selection in a population of advanced cycle winter wheat breeding lines. The poor predictive ability of LS for some traits results from the fact that complex traits are controlled by many QTL, thereby supporting the infinitesimal model of Fisher (1918) and the use of single-QTL models is naïve (Dekkers and Hospital 2002; Gianola 2006; Meuwissen et al. 2001). We also observed that when the trait was controlled by large effect loci, the benefits of LS over genomic prediction models was low. This was the case for seedling resistance to LR and YR in the 46th IBWSN and also APR to YR in several datasets in both nurseries. There were also some datasets in our study where LS performed slightly better than GBLUP. This can be attributed to the fact that LS may better capture large effect QTL and eliminate the noise due to the markers with near zero effect that are included in GBLUP. Hence, we would recommend using LS for oligogenic resistance and GBLUP for quantitative resistance. The GBLUP A model performed well for traits where the fixed effect markers explained a large amount of variation. A previous study by Rutkoski et al. (2014) for quantitative APR to SR in wheat also reported that GBLUP A gave higher accuracy than GBLUP alone. Although the average increase in accuracy using GBLUP A over GBLUP was only 1.3% in our study, it ranged between 15.4 and −36.6%.

Our results also indicate that the RKHS-M model performed similar to GBLUP, although several studies have reported that non-parametric models performed better than the parametric ones. Gianola (2006) used simulations and concluded that non-parametric RKHS model outperformed the parametric standard additive genetic model for additive by additive gene action. Crossa et al. (2010) reported that the RKHS models outperformed BLUP. Crossa et al. (2013) compared GBLUP with the RKHS and concluded that there was no clear superiority of either of the models, although the RKHS-M performed slightly better than the GBLUP. Howard et al. (2014) also reported that the non-parametric models performed well when the underlying genetic architecture was entirely based on epistasis. However, for the traits that we analysed in this study, either a negligible effect of epistasis or the equivalence of the RKHS-M to the GBLUP when the kernel used in RKHS is a Gaussian kernel (K = G) (Jiang and Reif 2015), led to similar accuracies.

We observed that RKHS-P performed well and the increase in accuracies using genome-wide marker-based models was only 4.44% (ranged between −20.94 and 38.2%). The pedigree-BLUP model was also evaluated and it gave similar accuracies as the RKHS-P model (results not shown). However, the general expectation is that the pedigree-based relationship would predict a 50% relationship between full-sibs and 25% relationship between half-sibs, while the genomic-based relationship would predict the allele sharing (within family variation) with better accuracy (Hayes and Goddard 2010). This is because it exploits the Mendelian sampling term that occurs during the formation of gametes and captures the realized relationship matrix instead of the average relationship matrix obtained from the pedigree (Daetwyler et al. 2007; Goddard and; Hayes 2007; Hayes et al. 2009; Villanueva et al. 2005). Crossa et al. (2010) reported that the gain in using markers compared to the pedigree was 7.7–35.7%. Wolc et al. (2011a) showed that marker estimated BVs were more persistent over generations compared to the pedigree estimated BVs in layer chickens. In another study, Wolc et al. (2011b) also reported that marker-based methods had higher accuracies than the pedigree based method. Spindel et al. (2015) reported that GS models were superior to the pedigree-based prediction in rice for yield, height, and flowering time. The benefits of using the G-matrix are manifold: (1) the G-matrix can differentiate sibs and can help avoid selecting closely related sibs together (Daetwyler et al. 2007); (2) the G-matrix can provide some prediction accuracies compared to the pedigree (almost zero) when distant/unrelated individuals are involved (van der Werf 2009); (3) the G-matrix can perform better when the pedigree is shallow (goes back to only a few generations); (4) the G-matrix can correct for pedigree errors (Munoz et al. 2014). Nevertheless, the fact that genotypes can also contain errors cannot be overlooked. We attribute the high accuracies obtained with the pedigree in our study to several reasons: (1) CIMMYT maintains an excellent pedigree recording system that goes back several generations. (2) The family sizes were small and except for large family sizes (with considerable Mendelian segregation), the advantage of using markers over the pedigree is expected to be small. (3) Dense marker coverage is essential to maximize the number of QTL that will be in LD with at least one marker that, in turn, is governed by the rate of decay of LD in the genome (Heffner et al. 2009). In this study, the large number of markers seem to provide excellent genome coverage. However, it is possible that these markers inadequately cover some major regions associated with the trait resulting in lower genomic prediction accuracies. (4) Another possibility is that, in the highly inbred lines we used, inbreeding resulted in the loss of alleles reducing the Mendelian sampling variance as suggested by Daetwyler et al. (2007). (5) Full-sibs in both the training and validation sets could have lead to higher accuracies with the pedigree, but this might not work as well for lines in early generations.

The RKHS-MP model performed better than just the pedigree and markers alone and gave the highest accuracies for most datasets which is consistent with several studies (Burgueño et al. 2012; Crossa et al. 2010, 2013; de los Campos et al. 2009; Perez et al. 2010). The average increase in accuracy using the RKHS-MP model over RKHS-P was 9.3% (ranged between −1.56 and 32.35%) and over the RKHS-M was 5.23% (ranged between −4.26 and 22.03%). Hence, despite, the pedigree being remarkably robust, it was clear that molecular markers can complement the pedigree to enhance breeding progress. Certain folds were predicted with a higher accuracy using the pedigree and vice versa, although the average accuracies were similar (data not shown). While it would be ideal to use both pedigree and markers to obtain the relationship matrix as suggested by Meuwissen (2007), consideration should be given to how informative the pedigrees are versus the cost of markers to make breeding decisions. However, there is a level of redundancy between the regression on the markers and that on the pedigree, and as a result, there might be only a small advantage of considering them together (Habier et al. 2009).

Although the IBWSNs were composed of a set of diverse lines involving several crosses between different parents, the ability to detect significant associations and predict resistance was not high in some datasets, especially where the resistance was quantitative. This was probably due to the lack of variability in these highly selected elite lines that resulted in low power. Hence, the issue is how to effectively implement genomic selection in later generations for traits with limited genetic variability. One strategy that can be applied to a large-scale breeding program is to develop a training population of a few hundred carefully chosen diverse fixed lines/varieties that vary widely for resistance to diseases of interest, are closely related to the breeding germplasm, and are grown in a managed nursery. These can be genotyped once and phenotyped for the desired diseases each season at a reasonable cost. In addition, new lines from the most recent germplasm can be added to the training population, so that prediction models for the highly selected late generation lines will provide more accurate results.

We observed that the loci significantly associated with seedling and APR were different for LR. But for YR, an all-stage resistance gene conferred resistance at both stages. This indicates a clear difference in the genetic basis for seedling resistance and APR to the two rusts in these populations. We also observed large differences between the different years/locations/replications for the traits. Several studies have focused on the incorporation of the genotype x environment (G × E) component in predictions (Burgueño et al. 2012; Heslot et al. 2013a, b; Jarquín et al. 2014; Lopez-Cruz et al. 2015) and it is important to consider the number of environments (years/locations/replications) that should be used for training the model, such that it is reasonably stable within and across environments. With whole-genome marker genotypes, the unit of replication is the allele and not the genotype *per se*. Therefore, using phenotyping strategies that can maximize the replication of alleles over the replication of individuals (Heslot et al. 2015) is important. In conclusion, our study clearly indicates that for quantitative traits, using genome-wide marker-based models maximizes genetic gain from molecular markers compared to marker-assisted selection. GS extends marker-assisted selection to a genome-wide scale and helps to make more accurate and informed breeding decisions for quantitative traits, thus advancing the revolution that molecular markers have brought to crop improvement.

### **Author contribution statement**

PJ drafted the manuscript and performed the research. MS, RPS, and PKS planned the study, supervised the analysis, and revised the manuscript. JC provided the pedigree data. JH, CL, and SB generated the phenotyping data. JR helped with the prediction models. JP performed the genotyping. GCB supervised the analysis and critically reviewed the manuscript.

## Electronic supplementary material

Below is the link to the electronic supplementary material.

Supplementary material 1 (DOCX 20 KB)

## Abbreviations

APR: Adult plant resistance
BLUP: Best linear unbiased prediction
BVs: Breeding values
CIMMYT: Centro Internacional de Mejoramiento de Maíz y Trigo
GBLUP: Genomic best linear unbiased prediction
GBLUP A: Genomic best linear unbiased prediction with selected loci as fixed effects
GBS: Genotyping-by-sequencing
HWWAMP: Hard winter wheat association mapping panel
IBWSN: International bread wheat screening nursery
IT: Infection type
KALRO: Kenya Agricultural and Livestock Research Organization
LD: Linkage disequilibrium
LR: Leaf rust
LS: Least squares
*Pt*: *Puccinia triticina*
*Pgt*: *Puccinia graminis*
*Pst*: *Puccinia striiformis*
QTL: Quantitative trait loci
RKHS-M: Reproducing kernel Hilbert spaces-markers
RKHS-MP: Reproducing kernel Hilbert spaces-markers and pedigree
RKHS-P: Reproducing kernel Hilbert spaces pedigree
RR-BLUP: Ridge regression-best linear unbiased prediction
SR: Stem rust
YR: Stripe rust
