# Supplementary material for: Genomic and pedigree-based prediction for leaf, stem, and stripe rust resistance in wheat
Source: Theor Appl Genet. 2017 Apr 9;130(7):1415–30. doi: 10.1007/s00122-017-2897-1 (PMC5487692; doi:10.1007/s00122-017-2897-1)
Supplement: Supplementary file 1 — Supplementary material 1 (DOCX 20 KB) [file 122_2017_2897_MOESM1_ESM.docx]

**Supplementary table 1** Basic local alignment search tool (BLAST) results for the significant markers in the 45^th^ and 46^th^ international bread wheat screening nursery (IBWSN)

| Marker | Subject^a^ | Score | Identities (Query length) | Percentage | Expect |
| --- | --- | --- | --- | --- | --- |
| GBS_24751 | gbsHWWAMP38350 | 113 | 63/64 (64) | 98 | 6.00E-23 |
|  | gbsCNLmaster31897 | 113 | 63/64 (64) | 98 | 6.00E-23 |
|  | 2BS_5218802 | 111 | 63/64 (64) | 98 | 2.00E-22 |
| GBS_37247 | WCSS1_contig470290_1DS-434 | 113 | 63/64 (64) | 98 | 6.00E-23 |
| GBS_8842 | WCSS1_contig3334901_3AS-4592 | 113 | 63/64 (64) | 98 | 6.00E-23 |
|  | synopGBS105165 | 111 | 62/63 (64) | 98 | 2.00E-22 |
| GBS_30281 | gbsHWWAMP47807 | 113 | 63/64 (64) | 98 | 6.00E-23 |
|  | WCSS1_contig7120458_4AL-2167 | 113 | 63/64 (64) | 98 | 6.00E-23 |
| GBS_22856 | WCSS1_contig10511286_3B-5360 | 113 | 63/64 (64) | 98 | 6.00E-23 |
| GBS_36529 | WCSS1_contig10759567_3B-1965 | 113 | 63/64 (64) | 98 | 6.00E-23 |
|  | synopGBS125790 | 111 | 62/63 (64) | 98 | 2.00E-22 |
| GBS_2454 | WCSS1_contig2284473_5BS-12686 | 114 | 63/63 (64) | 100 | 2.00E-23 |
|  | gbsHWWAMP2529 | 113 | 63/64 (64) | 98 | 6.00E-23 |
| GBS_13047 | gbsHWWAMP18106 | 113 | 63/64 (64) | 98 | 6.00E-23 |
| GBS_6432 | gbsHWWAMP7358 | 113 | 63/64 (64) | 98 | 6.00E-23 |
|  | WCSS1_contig5219749_2AS-4945 | 113 | 63/64 (64) | 98 | 6.00E-23 |
| GBS_702 | gbsHWWAMP627 | 113 | 63/64 (64) | 98 | 6.00E-23 |
|  | WCSS1_contig5304580_2AS-10182 | 109 | 62/64 (64) | 97 | 7.00E-22 |
| GBS_19971 | WCSS1_contig1905752_1DS-4628 | 114 | 63/63 (64) | 100 | 2.00E-23 |
| GBS_28186 | gbsHWWAMP44231 | 113 | 63/64 (64) | 98 | 6.00E-23 |
|  | gbsCNLmaster36568 | 113 | 63/64 (64) | 98 | 6.00E-23 |
|  | synopGBS121579 | 111 | 62/63 (64) | 98 | 2.00E-22 |
| GBS_28376 | WCSS1_contig1898017_1DS-2235 | 111 | 63/64 (64) | 98 | 2.00E-22 |
| GBS_40747 | gbsHWWAMP55344 | 113 | 63/64 (64) | 98 | 6.00E-23 |
|  | 2DS_5390754 | 116 | 64/64 (64) | 100 |  |
| GBS_18425 | gbsCNLmaster24464 | 116 | 64/64 (64) | 100 | 5.00E-24 |
|  | WCSS1_contig3419689_3AS-1090 | 116 | 64/64 (64) | 100 | 5.00E-24 |
| GBS_2400 | gbsHWWAMP2465 | 116 | 64/64 (64) | 100 | 5.00E-24 |
|  | WCSS1_contig3361063_3AS-2705 | 113 | 63/64 (64) | 98 | 6e-23 |
| GBS_1491 | gbsHWWAMP1393 | 116 | 64/64 (64) | 100 | 5.00E-24 |
| GBS_23856 | gbsHWWAMP37196 | 113 | 63/64 (64) | 98 | 6e-23 |
| GBS_28025 | WCSS1_contig3042477_6BS-5453 | 113 | 63/64 (64) | 98 | 6e-23 |
| GBS_20060 | WCSS1_contig2078323_6DS-22086 | 113 | 63/64 (64) | 98 | 6e-23 |

^a^ Marker prefixes and the population they were genotyped in or the map they are available in: gbsHWWAMP is for markers from the hard winter wheat association mapping panel; gbsCNLmaster is for markers from Cornell wheat master nursery; synopGBS is for markers in the Synthetic and Opata map (Poland et al., 2012) and WCSS1_contig is for markers in the CSS GBS 2014 physical map where ‘WCSS1’ stands for wheat chromosome survey sequence.
